# Economy in Scottish Hospitals

**Published:** 1905-03-18

**Authors:** 


### ECONOMY IN SCOTTISH HOSPITALS.

Colonel W. P. Warburton, M.D., Superintendent Royal Infirmary, Edinburgh, writes: A deputation from the London Hospital, consisting of the secretary, steward, and assistant matron, paid the Royal Infirmary, Edinburgh, a visit on January 19th to inquire into our arrangements and to discover if possible the secret of the economy practised here in connection with the question of administration recently raised by the commiteee of the King's Hospital Fund. I need scarcely say that no effort on our part was spared to help them in their quest. On February 4th a note appeared in the *British Journal of Nursing* in which the secretary of the London Hospital was credited with the opinion that the secret of the economy practised in the Scottish hospitals is fully explained by the nationality of the patients. The Scotsman will take porridge; the beef is boiled so that there is an abundance of soup. The Londoner, being more fastidious, will have none of these, and must have his beef roasted. As the above appeared to me to convey the incorrect impression that the patients in Scottish hospitals are only given porridge and boiled beef made into soup, I wrote to the secretary of the London Hospital drawing his attention to the note in question, which I quoted, and asked him if it was a fair summary of his opinion. He replied that the quotation from the *Journal of Nursing* was not correctly reported, or, rather, it was part but not all the truth. In acknowledging his letter I suggested that he should see the article in the *Journal of Nursing* and send a disclaimer to the journal if it did not represent his views. I have till now neither received any further communication from this gentleman nor have I seen any contradiction from him in the *British Journal of Nursing*; but in the London *Morning Post* of the 2nd inst. there was a notice of a meeting on the previous day of the governors of the London Hospital, presided over by Mr. Sydney Holland, who reported the result of the deputation which had visited certain northern hospitals with a view to seeing how it was that they were able to carry out their work at a lower cost per bed. He indicated a few items from one of the hospitals visited, which he named, which had a great advantage over the London Hospital. Meat in this hospital cost 3¾d. per pound, whereas in London it cost 4¾d. or 5d. They gave no medicines to their patients. Window cleaning cost £20 a year, against £300 in London, and no pensions were paid. As regards the question of cost per bed for provisions I was especially interested in the price paid for meat in the London Hospital, from which I had already obtained reliable information regarding four other articles of food—viz. milk, tea, coffee, and butter, as it enabled me to compare the cost of provisions per bed in the Royal Infirmary of Edinburgh with that of the London Hospital.

Meat in 1904 cost us a fraction over 6½d. per pound for home-fed Scottish beef and mutton, as compared with 4¾d. or 5d. per pound for foreign or colonial meat in the London Hospital. With us the difference of one farthing per pound in 1904 meant £194 6s. 2d. for the year, so that if we could have purchased our meat at 5d. instead of 6½d. there would have been a saving of £1,165 17s. For milk we paid 10¼d. per gallon in 1904, against 8¾d. paid by the London Hospital. The difference of one farthing per gallon in 1904 was equal to £80 18s. 9d. for the year, so that if we could have purchased it at 8¾d. we should have saved £485 12s. 6d. We give our patients tea, coffee, sugar, and butter; the London Hospital does not. The above-named six articles represent £11,152 1s. 2d., or 69 per cent. of the £16,157 13s. 5d. of our food expenditure in 1904, while poultry, fish, and eggs form an additional 14 per cent. On four days of the week patients who can eat it get roast meat (beef or mutton). On the other three days the meat is boiled, but the quantity issued is the same as on roast-meat days, and the fact that it is converted into Scotch broth means that there is a bigger equivalent of nourishment on those days. The cost of provisions per bed in the London Hospital was £31 5s. for 1903. In the Royal Infirmary for the same year it was £20 11s. 8¼d., and in 1904 it was £19 16s. 3d. I may here mention that our diet scale was made up and approved of by a committee of medical men, and that economy has been practised without in any way affecting efficiency. In 1904 we paid £273 5s. 7d. on account of pensions. The wages of our window-cleaning staff amounted to £226 12s. 8d. In 1901 we paid £365 4s. for window-cleaning. The reduction is due to improved methods in working. We give medicines to all our in-patients and supply dressings to all our surgical out-patients, who form 78 per cent. of the out-patients treated in this hospital in 1904. If investigations into the management of hospitals are considered necessary for the purpose of comparison I venture to suggest that they should be made by independent parties, who by comparing each item of expenditure in a number of hospitals would be in a position to judge where economies can be effected.

